# Reduced GABAergic Inhibition in the Basolateral Amygdala and the Development of Anxiety-Like Behaviors after Mild Traumatic Brain Injury

**DOI:** 10.1371/journal.pone.0102627

**Published:** 2014-07-21

**Authors:** Camila P. Almeida-Suhett, Eric M. Prager, Volodymyr Pidoplichko, Taiza H. Figueiredo, Ann M. Marini, Zheng Li, Lee E. Eiden, Maria F. M. Braga

**Affiliations:** 1 Program in Neuroscience, F. Edward Hébert School of Medicine, Uniformed Services University of the Health Sciences, Bethesda, Maryland, United States of America; 2 Department of Anatomy, Physiology and Genetics, F. Edward Hébert School of Medicine, Uniformed Services University of the Health Sciences, Bethesda, Maryland, United States of America; 3 Department of Neurology, F. Edward Hébert School of Medicine, Uniformed Services University of the Health Sciences, Bethesda, Maryland, United States of America; 4 Center for Neuroscience & Regenerative Medicine, F. Edward Hébert School of Medicine, Uniformed Services University of the Health Sciences, Bethesda, Maryland, United States of America; 5 Section on Clinical Studies, National Institute of Mental health Intramural Research Program, National Institutes of Health, Bethesda, Maryland, United States of America; 6 Section on Molecular Neuroscience, National Institute of Mental health Intramural Research Program, National Institutes of Health, Bethesda, Maryland, United States of America; McLean Hospital/Harvard Medical School, United States of America

## Abstract

Traumatic brain injury (TBI) is a major public health concern affecting a large number of athletes and military personnel. Individuals suffering from a TBI risk developing anxiety disorders, yet the pathophysiological alterations that result in the development of anxiety disorders have not yet been identified. One region often damaged by a TBI is the basolateral amygdala (BLA); hyperactivity within the BLA is associated with increased expression of anxiety and fear, yet the functional alterations that lead to BLA hyperexcitability after TBI have not been identified. We assessed the functional alterations in inhibitory synaptic transmission in the BLA and one mechanism that modulates excitatory synaptic transmission, the α_7_ containing nicotinic acetylcholine receptor (α_7_-nAChR), after mTBI, to shed light on the mechanisms that contribute to increased anxiety-like behaviors. Seven and 30 days after a mild controlled cortical impact (CCI) injury, animals displayed significantly greater anxiety-like behavior. This was associated with a significant loss of GABAergic interneurons and significant reductions in the frequency and amplitude of spontaneous and miniature GABA_A_-receptor mediated inhibitory postsynaptic currents (IPSCs). Decreases in the mIPSC amplitude were associated with reduced surface expression of α1, β2, and γ2 GABA_A_ receptor subunits. However, significant increases in the surface expression and current mediated by α_7_-nAChR, were observed, signifying increases in the excitability of principal neurons within the BLA. These results suggest that mTBI causes not only a significant reduction in inhibition in the BLA, but also an increase in neuronal excitability, which may contribute to hyperexcitability and the development of anxiety disorders.

## Introduction

Traumatic brain injury (TBI) is a major public health concern in the United States. There are approximately 1.8 million brain injuries annually [1], 90% of which may be classified as mild since patients do not display any clear morphological or functional abnormalities following injury [2], [3]. TBI is also a healthcare concern among athletes with approximately 300,000 cases per year [4], [5]. While most patients recover fully from mild TBI (mTBI), approximately 10–15% of patients have persistent cognitive, behavioral, and emotional complaints [6]–[9]. Of increasing concern is the prevalence of anxiety disorders after mTBI. Recent reports suggest that approximately 23% of individuals that sustain mTBI are at risk for developing anxiety disorders, and, particularly in the military, mTBI significantly increases the risk of developing posttraumatic stress disorder (PTSD) [10], [11].

Although clinical profiles of TBI are variable, mild and moderate TBI have been frequently localized to medial temporal lobe regions including the amygdala and are associated with long-term psychiatric symptoms [12]–[14]. The amygdala is a limbic structure deep within the temporal lobe that is involved in processing emotion and regulating behavioral and physiological responses to stressors [8], [15]. Amygdalar hyperactivity has been observed in the majority of functional neuroimaging studies investigating anxiety disorders [16]. In addition, after blast induced TBI, bilateral amygdalar hyperactivity has been observed in U.S. soldiers [17]. Thus, amygdalar dysfunction after a TBI, and in particular, neuronal hyperexcitability and hyperactivity in the basolateral nucleus of the amygdala (BLA), may be a key feature in the pathology of anxiety disorders, including PTSD in TBI victims [10], [18]–[21]. Although it is well established that anxiety disorders are more prevalent after mTBI, no study, to date, has identified the functional and morphological alterations that take place in the BLA underlying hyperexcitability after mTBI.

Increases in anxiety-like behavior are associated with the loss of GABAergic interneurons in the BLA [22] and reduced inhibitory synaptic transmission [23]. However, it remains unknown whether increased anxiety-like behavior after mTBI is due to deficits in GABAergic inhibition in the BLA. Therefore, we examined whether mTBI contributes to alterations in inhibitory synaptic transmission in the BLA. To this end, we have examined whether a mild controlled cortical impact (CCI) reduces GABA_A_ receptor mediated inhibitory postsynaptic currents (IPSCs) in the BLA and determined whether the loss of GABAergic inhibition is a result of interneuronal death or alterations in the surface expression of GABA_A_ receptors. In particular, we examined the α1, β2, and γ2 subunits, which comprise the majority of GABA_A_ receptor subtypes in the brain [24]. Because α_7_-containing nicotinic acetylcholine receptors (α_7_-nAChRs) are also present in the BLA and have previously been found to modulate BLA excitability [25], [26] and contribute to anxiety [27], we also examined whether α_7_-nAChR function and expression is altered in the BLA after mTBI. Our results suggest that mTBI increases anxiety-like behaviors, and that this may occur by injury-related deficits to inhibitory synaptic transmission. In addition, increased surface expression and ionic flow through α_7_-nAChRs on glutamatergic neurons may also contribute, in part, to hyperexcitability within the BLA and to long lasting increases in anxiety-like behavior.

## Experimental Procedures

### Ethics Statement

All animal experiments were conducted following the Guide for the Care and Use of Laboratory Animals (Institute of Laboratory Animal Resources, National Research Council) and were in accordance with the guidelines and approved by the Uniformed Services University of the Health Sciences Institutional Animal Care and Use Committees (IACUC). All efforts were made to minimize the number of animals used and any pain or distress associated with these experiments.

### Animals

Experiments were performed using 5–6 week old male, Sprague–Dawley rats (Taconic Farms, Rockville, MD, USA). Animals were housed in pairs until the day of the surgery and then housed individually in an environmentally controlled room (20–23°C, ∼44% humidity, 12-h light/12-h dark cycle [350–400 lux], lights on at 6:00 am), with food (Harlan Teklad Global Diet 2018, 18% protein rodent diet; Harlan Laboratories; Indianapolis, IN) and water available *ad libitum.* Cages were cleaned weekly and animal handling was minimized to reduce animal stress [28].

### Controlled Cortical Impact Injury

A unilateral cortical contusion using the controlled cortical impact (CCI) model of traumatic brain injury was administered using a previously established protocol [29]. Briefly, animals were anesthetized with isoflurane (2.5%) and had their heads shaved and placed in the stereotaxic frame. Core body temperature of the animals was maintained at 36–37°C using a heating pad and D.C. Temperature Control System (FHC, Bowdoin, ME). Without damaging the underlying dura mater, the skin was retracted, and a 4.0 mm craniotomy – 3.0 mm lateral to the midline and 4.0 mm posterior to the bregma over the left tempoparietal cortex – was performed. In these experiments, the contact velocity was set to 3.5 m/sec with a dwell time of 200 ms and the amount of deformation was set to 2.0 mm using a 3.0 mm diameter impact tip. These parameters have been shown to result in mild traumatic brain injury, i.e. causing no immediate trauma-induced cell death in areas contralateral to the impact, or even ipsilateral and subjacent to the cortical impact area [30]. Following injury, the skullcap was replaced and fixed using bone wax (Ethicon, Sommerville, NJ) and the incision was closed with absorbable sutures (Stoelting, IL). The animals received subcutaneously buprenorphine (50 µL) for pain alleviation and Ringer's solution (5 mL) for rehydration after surgery. Sham-treated controls received the craniotomy, but no CCI injury.

### Behavioral Analysis

#### Open Field Test

Anxiety-like behavior was assessed using an open field apparatus (40×40×20 cm clear Plexiglas arena) [31], [32] 2 days before and 1, 7, and 30 days after surgery. Animals were acclimated to the apparatus in the first session. On the test day, rats were placed in the center of the open field and activity was measured and recorded for 20 min, using an Accuscan Electronics infrared photocell system (Accuscan Instruments Inc., Columbus, OH). Data were automatically collected and transmitted to a computer equipped with “Fusion” software (from Accuscan Electronics, Columbus, OH). Locomotion (distance traveled in cm), total movement time, vertical activity, and time spent in the center of the open field were analyzed. Anxiety was measured as the ratio of the time spent in the center over the total movement time and expressed as a percentage of the total movement time, as previously described [32].

### Immunohistochemistry

#### Fixation and Tissue Processing

Seven (7) days after CCI, animals were deeply anesthetized using nembutal (75–100 mg/kg, i.p.) and transcardially perfused with phosphate buffered saline (PBS, 100 ml) followed by 4% paraformaldehyde (250 ml). Brains were removed and post-fixed in 4% paraformaldehyde overnight at 4°C, then transferred to a solution of 30% sucrose in PBS for 72 hours, and frozen with dry ice before storage at −80°C until sectioning. Sectioning was performed as previously described [33], [34]. A 1-in-5 series of sections containing the rostrocaudal extent of the amygdala was cut at 40 µm on a sliding microtome (Leica Microsystems SM2000R). A 1-in-5 series of free-floating sections was collected from the cryoprotectant solution, and washed three times for 5 min each. Slices were mounted on a slide, air-dried overnight and processed for Nissl staining with cresyl violet while the adjacent series of sections were mounted on slides for Fluoro-Jade C (FJC) staining.

#### GAD-67 Immunohistochemistry

To label GAD-67 immunoreactive neurons, a 1-in-5 series of free-floating sections was collected from the cryoprotectant solution, washed three times for 5 min each in 0.1 M PBS, and incubated in a blocking solution containing 10% normal goat serum (Millipore Bioscience Research Reagents, Temecula, CA) and 0.5% Triton X-100 in PBS for 1 hour at room temperature. The sections were then incubated with mouse anti-GAD-67 serum (1∶1000, MAB5406; Millipore Bioscience Research Reagents), 5% normal goat serum, 0.3% Triton X-100, and 1% bovine serum albumin overnight at 4°C. After rinsing three times for 10 min each in 0.1% Triton X-100 in PBS, the sections were incubated with Cy3-conjugated goat anti-mouse antibody (1∶1000; Jackson ImmunoResearch Laboratories Inc., West Grove, PA) and 0.0001% 4,6-diamidino-2-phenylindole dihydrochloride (Sigma-Aldrich) in PBS for 1 hour at room temperature. After a final rinse in PBS for 10 min, sections were mounted on slides, air-dried for at least 30 min, and coverslipped with ProLong Gold antifade reagent (Invitrogen, Carlsbad, CA).

#### Stereological Quantification

Design-based stereology was performed to quantify the total number of neurons in Nissl-stained sections and interneurons in GAD-67-immunostained sections in the BLA [34]. Sections were viewed with a Zeiss (Oberkochen, Germany) Axioplan 2ie fluorescent microscope with a motorized stage, interfaced with a computer running StereoInvestigator 8.0 (MicroBrightField, Williston, VT). The BLA was identified on slide-mounted sections and delineated for each slide of each animal, under a 2.5× objective, based on the atlas of Paxinos and Watson [35]. All sampling was done under a 63× oil immersion objective. Nissl-stained neurons were distinguished from glia cells by their larger size and pale nuclei surrounded by darkly stained cytoplasm containing Nissl bodies. The total number of Nissl-stained and GAD-67-immunostained neurons was estimated by using the optional fractionator probe, and, along with the coefficient of error (CE), were calculated by using StereoInvestigator 8.0 (MicroBrightField). The CE was calculated by the software according to the equations of Gundersen et al., (m = 1; [36]) and Schmitz and Hof (second estimation; [37]).

For Nissl-stained neurons in the BLA, a 1-in-5 series of sections was analyzed (six sections on average). The counting frame was 35×35 µm, the counting grid was 190×190 µm, and the dissector height was 12 µm. Nuclei were counted when the cell body came into focus within the dissector, which was placed 2 µm below the section surface. Section thickness was measured at every counting site, and the average mounted section thickness was 24.7 µm. An average of 187 neurons per hemisphere per rat were counted, and the average CE was 0.07 for both the Gunderson et al. and Schmitz-Hof equations.

For GABAergic interneurons immuno-labeled for GAD-67 in the BLA, a 1-in-5 series of sections was analyzed (on average six sections). The counting frame was 60×60 µm, the counting grid was 100×100 µm, and the dissector height was 20 µm. Nuclei were counted when the top of the nucleus came into focus within the dissector, which was placed 2 µm below the section surface. Section thickness was measured at every fifth counting site, and the average mounted section thickness was 24 µm. An average of 210 neurons per side per rat was counted, and the average CE was 0.08 for both the Gunderson et al. and Schmitz-Hof equations.

### Amygdala Slice Electrophysiology

Coronal slices containing the amygdala were prepared from rats 1 or 7 days after surgery. The rats were anesthetized with isofluorane and then decapitated. Coronal brain slices (400 µm-thick) containing the amygdala (−2.64 to −3.36 from Bregma) were cut using a vibratome (Leica VT 1200 S; Leica Microsystems, Buffalo Grove, IL), in ice-cold cutting solution consisting of (in mM): 115 sucrose, 70 NMDG, 1 KCl, 2 CaCl_2_, 4 MgCl_2_, 1.25 NaH_2_PO_4_, 30 NaHCO_3_, 25 D-glucose. Slices were transferred to a holding chamber, maintained at 32°C for 25 min, and then at room temperature, in a bath solution containing (in mM): 125 NaCl, 2.5 KCl, 2.0 CaCl_2_, 2.0 MgCl_2_, 21 NaHCO_3_, 1.25 NaH_2_PO_4_, and 22 D-glucose. Recording solution was the same as the holding bath solution. All solutions were saturated with 95% O_2_, 5% CO_2_ to achieve a pH near 7.4. Slices were transferred to a submersion-type recording chamber (0.7 mL capacity), where they were continuously perfused with oxygenated ACSF (flow rate about 5 mL/min). Neurons were visualized with an upright microscope (Zeiss Axioskop 2, Thronwood, NY) through a 40× water immersion objective, equipped with a CCD-100 camera (Dage-MTI, Michigan City, IN). All experiments were performed at 32°C. Tight-seal (>1 GΩ) whole-cell recordings were obtained from the cell body of pyramidal-shaped neurons in the BLA region, which were identified on the basis of their electrophysiological properties [38], [39]. Current activated by hyperpolarization (I_h_ current) characteristic of principal neurons was recorded during the first minutes after breaking into the cell. Patch electrodes were fabricated from borosilicate glass and had a resistance of 3.5–4.5 MΩ when filled with solution A containing (in mM): 135 Cs-gluconate, 10 MgCl_2_, 0.1 CaCl_2_, 1 EGTA, 10 Hepes, 2 Na–ATP, 0.2 Na_2_GTP, pH 7.3 (285–290 mOsm) or solution B containing (in mM): 60 Cs-gluconate, 60 KCH_3_SO_3_, 10 KCl, 10 EGTA, 10 HEPES, 5 Mg-ATP, 0.3 Na_2_GTP, pH 7.2 (280–290 mOsm/kg). Solution A was used to record spontaneous and miniature inhibitory postsynaptic currents (IPSCs) and solution B was used in experiments involving bath application of choline. Neurons were voltage-clamped using an Axopatch 200B amplifier (Axon Instruments, Foster City, CA, USA). IPSCs were pharmacologically isolated and recorded at a −70 mV holding potential as inward currents in voltage-clamp mode. Access resistance (15–24 MΩ) was regularly monitored during recordings, and cells were rejected if it changed by 15% during the experiment. Ionic currents and action potentials were amplified and filtered (1 kHz) using the Axopatch 200B amplifier (Axon Instruments, Foster City, CA) with a four-pole, low-pass Bessel filter, were digitally sampled (up to 2 kHz) using the pClamp 10.2 software (Molecular Devices, Sunnyvale, CA), and further analyzed using the Mini Analysis program (Synaptosoft Inc., Fort Lee, NJ) and Origin (OriginLab Corporation, Northampton, MA). The peak amplitude, 10–90% rise time, and decay time constant of IPSCs were analyzed off-line using pClamp 10.2 software and the Mini Analysis Program (Synaptosoft, Inc., Leonia, NJ, USA). Miniature IPSCs (mIPSCs) were analyzed off-line using the Mini Analysis Program (Synaptosoft, Fort Lee, NJ) and detected by manually setting the mIPSC threshold (∼1.5 times the baseline noise amplitude) after visual inspection.

Agonists of α_7_-nAChRs were applied by pressure injection. Pressure application was performed with the help of a push–pull experimental arrangement [40], as utilized previously [33]. Pressure was applied to the pipette via a Picrospritzer (General Valve Division, Parker Hannifin Corp., Fairfield, NJ), set at about 100 MPa (14 psi). A motorizer (Newport, Fountain Valley, CA) was coupled with the approach/withdrawal (push–pull) actuator of a micromanipulator (Burleigh PCS-5000 series; EXFO Photonic Solution Inc., Mississauga, Ontario, Canada). Motorizer movement and duration of application pulses were controlled with a Master-8 digital stimulator (AMPI; Jerusalem, Israel). Ionic currents were amplified and filtered (1 kHz) using an Axopatch 200B amplifier, with a four-pole low-pass Bessel filter, and were digitally sampled (up to 5 kHz). Currents were recorded using pClamp 10.2 software and further analyzed using OriginLab (Northampton, MA) and Mini60 software.

Drugs used were as follows: 20 µM 6-cyano-7-nitroquinoxaline-2,3-dione (CNQX), an α-amino-3-hydroxyl-5-methyl-4-isoxazole-propionate (AMPA)/kainate receptor antagonist; 50 µM d-2-amino-phosphonovalerate (AP-5), an *N*-methyl-d-aspartic acid (NMDA) receptor antagonist; 10 µM SCH50911, a GABA_B_ receptor antagonist, 3 µM LY341495, a metabotropic group II/III glutamate receptor antagonist, and 1 µM α-conotoxin Au1B, an α_3_β_4_-nicotinic receptor antagonist (all purchased from Tocris, Ellisville, MO). We also used 20 µM bicuculline methiodide, a GABA_A_ receptor antagonist, 1 µM tetrodotoxin (TTX), a sodium channel blocker, 10 µM dihydro-β-erythroidine (DHβE), an α_4_β_2_-nicotinic receptor antagonist; 5 mM tricholine citrate, an α_7_-nicotinic receptor agonist, and 0.5 µM atropine sulfate, a muscarinic AChR antagonist (purchased from Sigma-Aldrich, St. Louis, MO).

### Biotinylation and Western Blot

Coronal slices containing BLA were prepared as described for electrophysiology experiments. After a 1-hour recovery period in oxygenated ACSF, slices were incubated in ACSF containing 1 mg/ml EZ-Link Sulfo-NHS-SS-Biotin (Pierce, Rockford, IL) for 1 hour on ice, followed by the addition of quench solution (provided in the Pierce Cell Surface Protein Isolation Kit, Cat No. 89881). The BLA was then dissected and tissue sections were transferred to small plastic tubes containing radioimmunoprecipitation assay (RIPA) buffer composed of (in mM) 50 Tris-HCl, pH 7.4, 150 NaCl, 2 EDTA, 50 NaF, 1 Na_3_VO_4_, 1% Triton X-100, 0.1% SDS, 0.5% Na-deoxycholate, and a Protease Inhibitor Cocktail (Sigma-Aldrich, MO). Samples were sonicated and the homogenates were centrifuged at 14,000 g for 10 min at 4°C. Protein concentrations were measured using the DC Protein Assay Kit (Bio-Rad, CA). Protein (1,500 µg) was then mixed with 400 µL of UltraLink immobilized NeutrAvidin agarose beads (Pierce) for 1 hour at room temperature. The beads were then washed 3 times with 500 µL wash buffer (provided in the kit). Samples were eluted in 400 µL of RIPA buffer containing Protease Inhibitor Cocktail supplemented with 50 mM dithiothreitol and mixed for 1 hour at room temperature followed by centrifugation at 14,000 g for 10 min at 4°C. Then, LDS 4× (Invitrogen) was added to protein samples. Biotinylated proteins were resolved by SDS-PAGE, transferred to nitrocellulose membranes, and probed with the following antibodies Anti-Alpha1 GABA-A Receptor, clone N95/65, 75–136 (1∶1000; UC Davis/NIH NeuroMab Facility), Anti-GABA A Receptor Beta 2,3 Chain, clone BD17|MAB341 (1∶1000, Millipore, Billerica, MA), Anti-GABA A Receptor Gamma 2, Ab16213 (1∶1000, AbCam, Cambridge, MA), Anti-Nicotinic Acetylcholine Receptor (α7 Subunit) M220 (1∶1000, Sigma-Aldrich, St. Louis, MO). The signal from the immunoreactive band was detected by using a gel imaging system (Fuji LAS-3000). Membranes were stripped using ReBlot Plus Strong Antibody Stripping Solution (Millipore, Billerica, MA) and re-probed with Anti-GLUT1 (1∶1000, Millipore, Billerica, MA) for loading control. Signal intensity was determined by densitometric scanning using ImageJ. When duplicate conditions were performed in one animal, the ratio values were averaged to obtain an animal average for that condition.

### Statistical Analysis

Statistical values are presented as mean ± standard error (SE). Results from ipsilateral and contralateral sides of sham-operated and traumatized animals were compared using one-way ANOVA followed by Bonferroni post-hoc test in the stereology and Western blot experiments. For open-field experiments, mixed design ANOVA followed by independent t-tests were used. For electrophysiology experiments, either one-way ANOVA followed by Bonferroni post-hoc test or independent t-tests were performed. p<0.05 was considered statistically significant for all statistical analysis. Sample sizes (n) refer to the number of rats, except for the electrophysiology results where “n” refers to the number of slices or recorded cells.

## Results

### Anxiety-like behaviors increase 7 days after CCI

**Figure 1:**
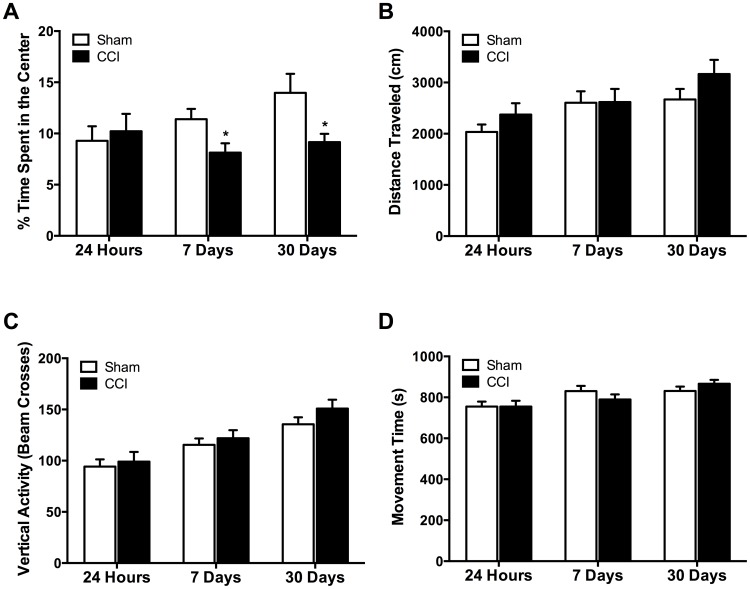
Mild TBI increases anxiety-like behaviors in the open field test. (A) No differences in percent time spent in the center of the open field were found between CCI (n = 19) and sham (n = 19) animals 24 hours after injury. However, CCI rats spent significantly less time in the center of the open field 7- and 30-days after injury compared to sham animals. No significant differences were found between the sham and CCI animals in distance traveled (B), vertical activity (C), or movement time (D) at any of the time points. Bars show the mean ± SE of the percentage of time spent in the center (A), distance traveled (B), vertical activity (C), and movement time (D). *p<0.05.

Rats exposed to mild CCI were tested in the open field apparatus 2 days before and 1, 7 and 30 days after surgery. Overall, mTBI caused a significant increase in anxiety-like behavior, as tested by the percent time spent in the center of the open field (F(1,36) = 4.14; p = 0.049). Prior to surgery, there were no differences between sham (8.7±1.2% of the total movement time; n = 19) and CCI (5.8±0.8% of the total movement time; n = 19) groups in the percent time spent in the center of the open field, distance traveled (2,003.4±131.3 cm for sham animals, 2,003.4±157.0 cm for CCI animals), vertical activity (105.6±7.2 for sham animals, 96.8±7.1 beam crosses for CCI animals), or movement time (761.1±20.9 s for sham animals, 742.7±22.8 s for CCI animals; p's>0.05). One day after the surgery, sham (9.3±1.4% of the total movement time) and CCI (10.2±1.7% of the total movement time) animals also spent similar amount of time in the center of the field (p = 0.68; Figure 1A). CCI animals did not differ from sham animals in distance traveled (2,034.9±144.7 cm for sham animals and 2,373.7±221.1 cm for CCI animals; Figure 1B), vertical activity (94.2±7.1 beam crosses for sham animals, 99.1±9.4 beam crosses for CCI animals; Figure 1C), or movement time (754.9±24.5 sec for sham animals, 754.9±28.2 sec; p's>0.05; Figure 1D). However, 7 days post injury, CCI rats (8.1±0.9% of the total movement time) spent significantly less time in the center of the open field compared to sham animals (11.4±1.0% of the total movement time; p = 0.022). CCI animals did not differ from control animals in distance traveled (2,605.3±224.0 cm for sham animals, 2,618.0±256.7 cm for CCI animals), vertical activity (115.5±6.2 beam crosses for sham animals, 121.9±7.9 beam crosses for CCI animals), or movement time (830.9±25.2 sec for sham animals, 789.6±24.6 sec for sham animals; p's>0.05; Figure 1). Anxiety-like behavior was also observed 30 days post injury as CCI rats (9.2±0.8% of the total movement time) spent significantly less time in the open field compared to sham animals (14.0±1.9% of the total movement time; p = 0.024; Figure 1). CCI animals did not differ significantly from control animals in distance traveled (2,670.3±204.9 cm for sham animals, 3,165.8±276.4 cm for CCI animals), vertical activity (135.6±6.8 beam crosses for sham animals, 150.9±8.7 beam crosses for CCI animals), or movement time (831.2±21.5 sec for sham animals, 866.6±18.7 sec for sham animals; p's>0.05).

### Neuropathology of Principal Neurons and Interneurons in the BLA after CCI

**Figure 2:**
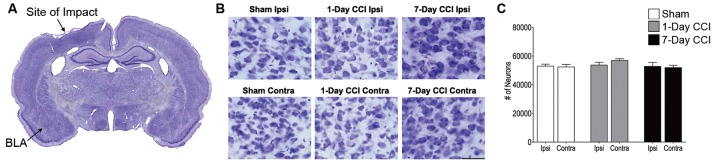
Mild CCI does not cause a significant loss of neurons in the BLA 24 hours or 7 days after injury. (A) Panoramic photomicrograph of Nissl-stained brain slice. Indicated are the sites of impact and the ipsilateral BLA. (B) Representative photomicrographs from Nissl-stained sections showing BLA cells from the ipsilateral (Top) and contralateral (Bottom) sides of sham, 1-day CCI, and 7-day CCI animals, respectively. Total magnification is 630X; scale bar, 50 µm. (C) Group data (mean ± SE; n = 8 for each group) of stereological estimation of the total number of Nissl-stained neurons in the BLA.

**Figure 3:**
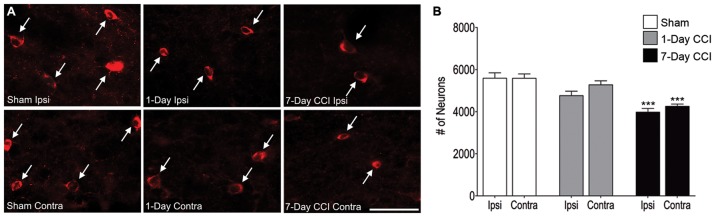
Delayed loss of GABAergic interneurons in the BLA within the first week after mild CCI. (A) Representative photomicrographs of GAD-67 immunohistochemically stained GABAergic interneurons in the BLA of sham (left), 1-day CCI (middle), and 7-day CCI (right) animals. Total magnification is 630x; scale bar, 50 µm. (B) group data showing the mean and standard error of the stereologically estimated total number of GAD-67-positive cells in the BLA 1- and 7-days after CCI compared with sham. Only 7-days after CCI was there a significant bilateral reduction in GAD-67-positive cells indicating a delayed loss of GABAergic interneurons. ***p<0.001; n = 10 for each group.

We next examined whether the increase in anxiety-like behavior was associated with a loss of neurons and interneurons after CCI. Estimation of the total number of neurons in the BLA, using an unbiased stereological method in Nissl-stained sections, revealed that animals that received a CCI did not lose a significant number of total neurons 1- or 7-days after injury compared to age-matched sham injured control animals (Table 1; Figure 2). We next asked whether CCI induced interneuronal death 1- or 7- days after injury, as previous studies have found a significant loss of interneurons after mTBI [41]. Estimation of the total number of interneurons in the BLA, using an unbiased stereological method to quantify GAD67 immunoreactive cells, showed that there was no significant loss of interneurons 1 day after CCI either ipsilateral or contralateral to the site of injury compared to sham controls (Table 2; Figure 3). Similar to the Nissl-stained sections, GAD67-positive cells from 1- and 7-day sham control groups did not display any significant differences and were therefore averaged together (data not shown). However, 7 days after CCI, animals showed a 28.8% reduction ipsilateral and a 23.8% reduction in GAD67-positive cells contralateral to the site of injury, indicating a significant loss in the number of GAD67-positive neurons in the BLA (Table 2; Figure 3B).

**Table 1:** Total Number of Neurons.

Condition	# of Cells ± SEM	Compared to	# of Cells ± SEM
**Sham Ipsilateral**	52,969±1,392	CCI 1 day Ipsilateral	52,815±2,723
		CCI 1 day Contralateral	51,961±1,608
		CCI 7 days Ipsilateral	53,733±2,221
		CCI 7 days Contralateral	56,922±1,451
**Sham Contralateral**	52,400±1,741	CCI 1 day Ipsilateral	52,815±2,723
		CCI 1 day Contralateral	51,961±1,608
		CCI 7 days Ipsilateral	53,733±2,221
		CCI 7 days Contralateral	56,922±1,451

**Table 2:** Total Number of Interneurons.

Condition	# of Cells ± SEM	Compared to	# of Cells ± SEM
**Sham Ipsilateral**	5,550±262	CCI 1 day Ipsilateral	4,763±2010
		CCI 1 day Contralateral	5,277±190
		CCI 7 days Ipsilateral	3,980±172***
		CCI 7 days Contralateral	4,254±107**
**Sham Contralateral**	5,702±208	CCI 1 day Ipsilateral	4,763±2010
		CCI 1 day Contralateral	5,277±190
		CCI 7 days Ipsilateral	3,980±172***
		CCI 7 days Contralateral	4,254±107**

**p<0.01, ***p<0.001.

### Alterations in GABA_A_-mediated spontaneous and miniature IPSCs

Whole-cell recordings were obtained from BLA neurons that were identified on the basis of their size, pyramidal-like shape, firing patterns in response to depolarizing current pulses in the current-clamp mode, and the presence of a current activated by hyperpolarizing voltage-steps (*I_h_*), in the voltage clamp mode. Depolarizing current injections generated variable patterns of accommodating spiking. Four 1 s-long hyperpolarizing pulses starting from V_hold_ −70 to −80 mV and ending with −110 mV elicited nonlinear I*_h_* current in principal neurons [32], [42] (data not shown).

**Figure 4:**
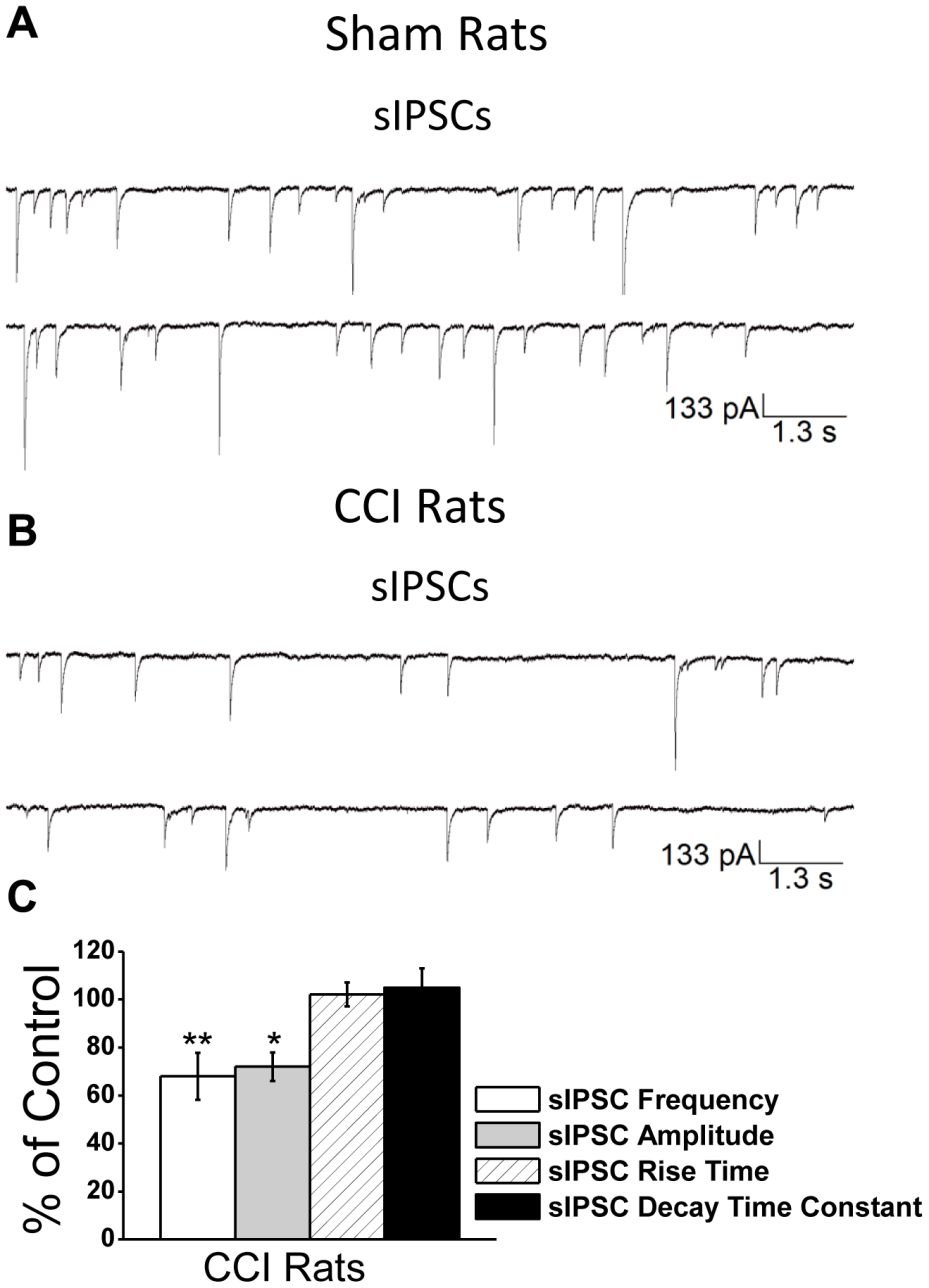
Mild CCI causes a significant decrease in the frequency and amplitude of sIPSCs in the BLA 7-days after CCI. sIPSCs were recorded from pyramidal-shaped neurons in the presence of CNQX, D-AP5, SCH50911, and LY 3414953, at a holding potential of −70 mV. Representative examples of recordings obtained in the BLA are shown in (**A**) and (**B**) for Sham and CCI 7-day animals, respectively. (**C**) Group data showing the change in the percentage frequency and amplitude of sIPSCs from CCI animals relative to Sham animals. The frequency and amplitude, but not the rise time and the decay time constant of the sIPSCs were significantly reduced in the CCI group compared to the sham controls. *p<0.05; **p<0.01; n = 18 for each group.

To determine whether CCI impaired inhibitory synaptic transmission, we recorded spontaneous GABA_A_ receptor-mediated IPSCs (sIPSCs) from principal neurons in the presence of CNQX, D-AP5, SCH50911, and LY 3414953 at a holding potential of −70 mV. Sham animals did not differ in the frequency and amplitude of sIPSCs at either 1- or 7-days after surgery and so we averaged together the amplitude and frequency of all sham animals. We examined the frequency and amplitude of GABA_A_ receptor-mediated sIPSCs 1- and 7-days after CCI. Compared to sham animals (mean frequency  = 2.9±0.7 Hz; mean amplitude  = 288±9 pA; n = 18), we found no significant difference in the frequency (2.7±1.2 Hz; n = 18; *p>*0.05) and amplitude (293±12 pA; n = 18; *p>*0.05) of GABA_A_ receptor-mediated sIPSCs 1 day after CCI. However, 7 days after CCI we found a 32±9.8% reduction in the frequency (1.8±0.5 Hz; n = 18; *p<*0.01) and a 28±5.9% reduction in the amplitude (211±8 pA; n = 18; *p<*0.05) of GABA_A_ receptor mediated sIPSCs (Figure 4).

**Figure 5:**
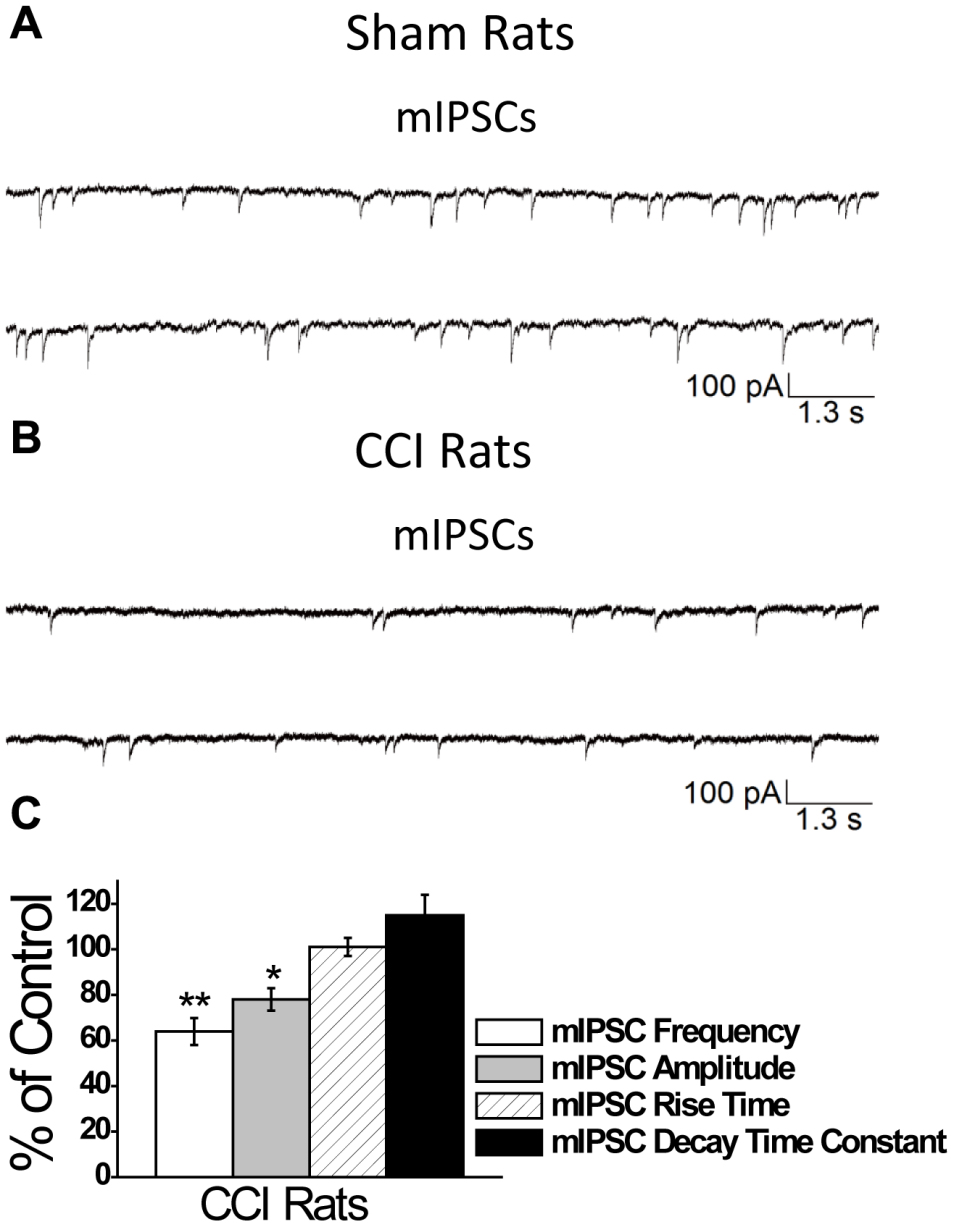
Mild CCI causes a significant decrease in the frequency and amplitude of mIPSCs in the BLA 7-days after CCI. mIPSCs were recorded from pyramidal-shaped neurons in the presence of CNQX, D-AP5, SCH50911, LY 3414953, and TTX at a holding potential of –70 mV. Representative examples of recordings obtained in the BLA are shown in (**A**) and (**B**) for Sham and CCI 7 day animals, respectively. (**C**) Group data showing the change in the percentage frequency and amplitude of mIPSCs from CCI animals relative to Sham animals. The frequency and amplitude, but not the rise time and the decay time constant of the sIPSCs were significantly reduced in the CCI group compared to the sham controls. The recorded currents were blocked by the GABA_A_ receptor antagonist bicuculline (data not shown). *p<0.05; **p<0.01; n = 17 for each group.

To determine how CCI impaired inhibitory transmission, we examined the effects of CCI on miniature IPSCs (mIPSCs), recorded in the presence of CNQX, D-AP5, SCH50911, and LY 3414953, and TTX at a holding potential of −70 mV. Recording mIPSCs from principal neurons in the BLA allows us to directly examine whether changes in the probability of quantal release at the presynaptic terminal or internalization of postsynaptic GABA_A_ receptors contributed to impaired inhibitory synaptic transmission. Sham animals did not differ in the frequency and amplitude of mIPSCs at either 1- or 7-days after surgery and so we averaged together the amplitude and frequency of all sham animals (data not shown). We examined the frequency and amplitude of GABA_A_ receptor-mediated mIPSCs 1- and 7-days after CCI. Compared to sham animals (mean frequency  = 1.5±0.7 Hz; mean amplitude  = 59±8 pA; n = 17), we found no significant difference in the frequency (1.5±0.4 Hz; n = 17; *p*>0.05) and amplitude (55±10 pA; n = 17; *p*>0.05) of GABA_A_ receptor-mediated mIPSCs 1 day after CCI. However, 7 days after CCI we found a 35±5% reduction in the frequency (0.9±0.5 Hz; n = 17; *p<*0.01) and a 22±4.8% reduction in the amplitude (44±9 pA; n = 17; *p<*0.05) of GABA_A_ receptor mediated mIPSCs (Figure 5).

### Reduced membrane expression of GABA_A_ receptor subunits

**Figure 6:**
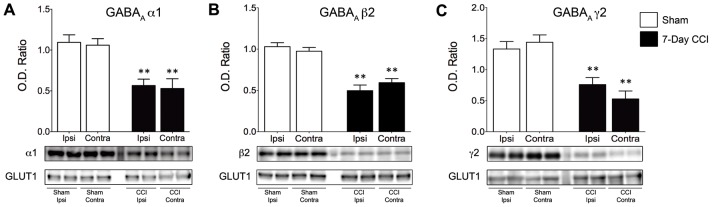
Surface expression of α1, β2, and γ2 GABA_A_ receptor subunits is reduced in the BLA of CCI animals 7 days after mild CCI. Western blot for subunits of (A) α1, (B) β2, and (C) γ2 subunits, respectively, was performed using biotinylated proteins isolated from the ipsilateral and contralateral sides of Sham and CCI 7-day animals. Group data showing the mean ± SE of the ratio between each subunit and GLUT1 optical densities. Top panel: representative Western blot for α1 (A), β2 (B), and γ2 (C) subunits of GABA_A_ receptors, respectively. Bottom panel: representative Western blot for GLUT1, used as a loading control. Note that surface expression of GABA_A_ α1, β2 and γ2 subunits are reduced in CCI animals when compared to Sham animals. *p<0.01; n = 4 for each group.

Because we found a decrease in both the amplitude and frequency of GABA_A_ receptor-mediated IPSCs, the latter of which may be a result of the significant loss of interneurons observed 7 days after CCI, we next examined whether reduced surface expression of the GABA_A_ receptor contributed to the reduced amplitude of mIPSCs. To determine whether the surface expression of GABA_A_ receptor subunits was reduced after CCI, we examined alterations to the α1, β2, and γ2 subunits from the BLA, subunits that constitute the majority of GABA_A_ receptors in the brain and are highly expressed in the BLA [24]. Membrane proteins were isolated by biotinylation assay and levels of specific subunits were quantified by Western blot. After densiometric analysis, Western blot membranes were stripped and re-probed for GLUT1. It has been previously demonstrated that expression of GLUT1 is unaltered following TBI [43] and therefore was used as a loading control. We found that the surface expression of all three GABA_A_ subunits were reduced 7 days (n = 4) after CCI both ipsilateral (47.3%; *p*<0.001) and contralateral to the site of impact (52.5%; *p*<0.001) compared to sham control animals (n = 4). Ipsilateral to the side of impact, a 48.3% (*p* = 0.002) reduction of the α1 subunit, a 51.8% (*p*<0.001) reduction of the β2 subunit, and a 43.1% (*p*<0.001) reduction of the γ2 subunit, were found, whereas contralateral to the side of impact, 50.2% (*p*<0.001) reduction of the α1 subunit, a 47.3% (*p*<0.001) reduction of the β2 subunit, and a 60.3% (*p*<0.001) reduction of the γ2 subunit, was observed, indicating that the reduced amplitude in the GABA_A_ receptor-mediated sIPSCs may be due to reductions in the surface expression of GABA_A_ receptors (Figure 6).

### Alterations in α_7_-nAChR-mediated currents after mTBI

**Figure 7:**
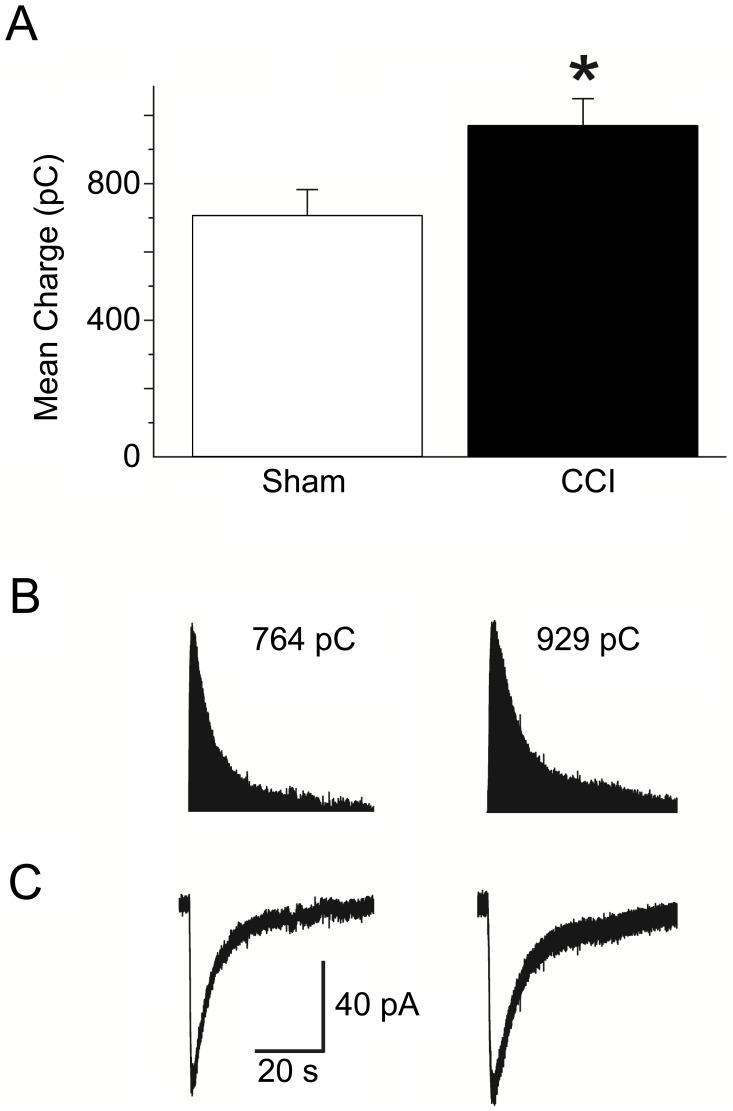
Activation of α7-nAChRs by fast application of the α_7_-nAChR agonist tricholine citrate, in the BLA, shows increased cholinergic conductance 7-days after CCI. (A) Group data showing the mean ± SE charge transfer in pyramidal-shaped neurons in the BLA from CCI rats 7-days after injury (970±80 pC; n = 17) was significantly increased compared to sham rats (715±66 pC; n = 16). (B) Representative charge transfer from α_7_-nAChRs from sham (left), and 7-day CCI (right) animals. Note the increase in decay through α7-nAChRs in the BLA at day 7 post injury. (C) Representative α_7_-nAChR-mediated currents recorded from sham (left), and 7-day CCI (right) animals. The increase in the decay and amplitude of the α_7_-nAChR-mediated current 7-days post CCI led to increases in the charge transferred through by α_7_-nAChRs. Experiments were recorded in the presence of α-conotoxin Au1B, DHβE, atropine sulfate, D-AP5, CNQX, SCH50911, LY 3414953, and bicuculline. *p<0.05;

In the BLA, α_7_-nAChRs have previously been reported to be present on somatodendritic regions of glutamatergic neurons [25] and are involved in presynaptically facilitating glutamate release [26], [44]. To determine whether α_7_-nAChRs contributed to the principal cell hyperexcitability, we pressure-applied tricholine citrate (5 mM; 70 ms; 14 psi), while recording from principal neurons of sham control slices (n = 17) in the presence of α-conotoxin Au1B, DHβE, atropine sulfate, D-AP5, CNQX, SCH50911, LY 3414953, and bicuculline and examined the mean charge transferred, a measurement that reflects the amount of current (charged particles) flowing into the cell by integrating the duration of the open time of ion channels. In current-clamp mode, pressure application of tricholine citrate onto control slices elicited a brief train of action potentials, while in the voltage clamp mode it induced inward currents. The current induced by tricholine citrate was nearly blocked by 1 µM α-BgTx. The mean charge transferred through α_7_-nAChRs in sham animals was 715±66 pC (n = 16). 1-day after CCI we did not observe any alteration in the mean charge transferred through α_7_-nAChRs (748±63 pC; n = 16). However 7-days after CCI, the mean charge transferred was significantly increased (970±80 pC; n = 17) compared to sham animals. Puff application of tricholine citrate elicited α_7_-nAChR currents that were 35.6% (p = 0.035) larger in 7-day CCI compared to sham animals, suggesting an increase in the membrane expression of functional α_7_-nAChRs (Figure 7).

### Increased membrane expression of α_7_-nAChRs after CCI

**Figure 8:**
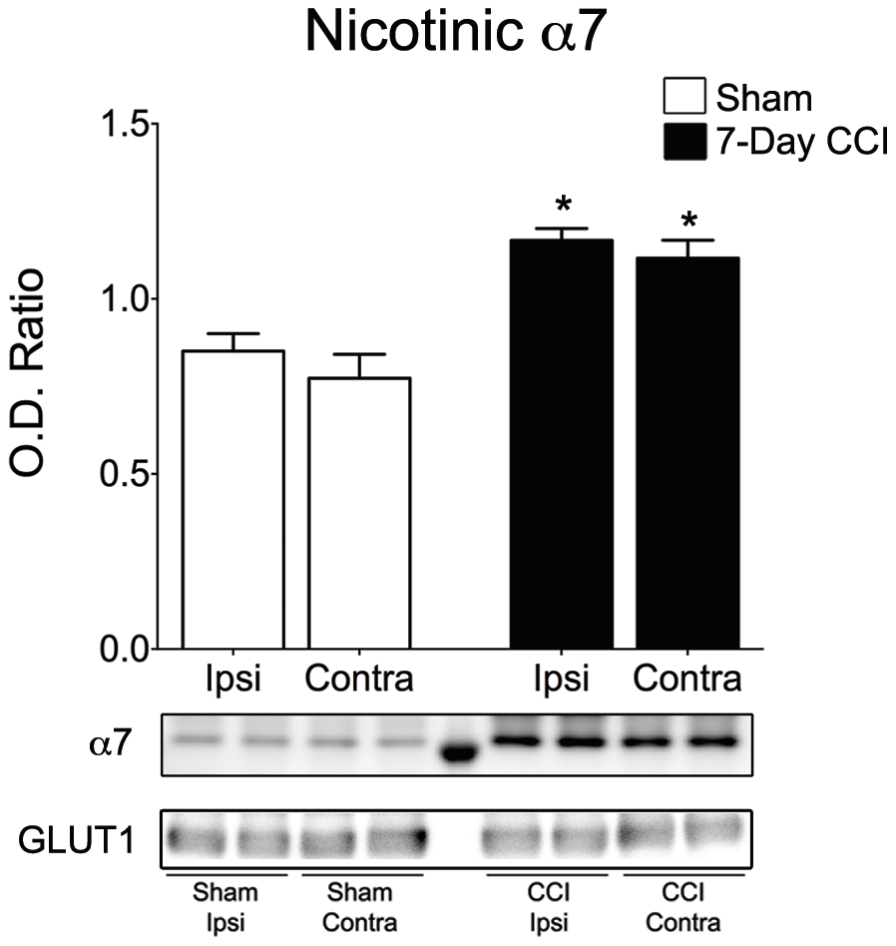
Surface expression of α7 subunit of neuronal nicotinic acetylcholine receptor is increased in the BLA of CCI animals 7-days after mild CCI. Western blot was performed using biotinylated proteins isolated from the ipsilateral and contralateral sides of Sham and CCI 7-day animals. Group data showing the mean ± SE of the ratio between the α7 subunit and GLUT1 optical densities. Top panel: representative Western blot for α_7_-nAChRs. Bottom panel: representative Western blot for GLUT1, used as a loading control. *p<0.05; n = 4 for each group.

We used biotinylation and Western blot analysis to determine whether the surface expression of α_7_-nAChRs increase after CCI and contributed to the 35.6% increase in the charge transferred by α_7_-nAChRs. We found that the surface expression of the α_7_-nAChR was increased by 37.2% (*p*<0.001) 7 days after CCI (n = 4) compared with sham control animals (n = 8). In CCI animals the increase in the surface expression of α_7_-nAChRs was 44.2% (*p* = 0.017) on the contralateral site of injury versus 37.2% (*p* = 0.049) ipsilateral to the site of injury (Figure 8).

## Discussion

The present study revealed that CCI-induced mTBI caused a long-lasting increase in anxiety-like behaviors, as tested by the open field. This was accompanied by, and may be the result of reduced GABA_A_ receptor mediated inhibitory synaptic transmission and increased excitability within the BLA. Reduced inhibitory tonus within the BLA is consistent with a delayed but significant loss of interneurons as compared to the total number of neurons within the BLA, and a significant decrease in the surface expression of α1, β2, and γ2 GABA_A_ receptors subunits after mTBI. Interneuronal loss and reductions in the surface expression of GABA_A_ receptors led to a reduction in the frequency and amplitude of GABA_A_-receptor mediated spontaneous and miniature IPSCs, respectively. Additionally, in principal neurons, we observed a significant increase in the charge transferred by α_7_-nAChRs. The increase in charge transferred via the α_7_-nAChRs was accompanied by an increase in surface expression of α_7_-nAChRs in the BLA. Together, reductions in GABAergic inhibitory transmission and an increase in α_7_-nAChR function, may contribute, in part, to hyperexcitability in the BLA and long-lasting increases in anxiety-like behavior observed after mTBI.

Long-lasting increases in anxiety and the development of anxiety disorders have been consistently observed in humans following TBI [45]–[47]. The recent focus of media attention on sports related injuries has revealed that mild concussions sustained during play increases the incidence of developing neuropsychiatric disorders, including anxiety disorders [48], [49]. Similarly, U.S. soldiers exposed to TBI are significantly more likely to report anxiety and anxiety disorders compared to soldiers that did not suffer a TBI [10]. Accordingly, anxiety-like behaviors have been observed in animals in the present study, and using different models of TBI [11], [50], [51], including fluid percussion injury [50], blast exposure [51], and mTBI induced by CCI [11]. Thus, mTBI may cause functional and morphological alterations in the BLA underlying long-lasting increases in anxiety-like behavior in animals and the manifestation of anxiety disorders in humans.

Although it is apparent that mild TBI increases the prevalence of developing anxiety disorders [52], it has not been excluded that the anxiety may be due to the stress associated with the trauma and its aftermath and not with trauma itself [8], [10], [15], [53]. Here, we aimed to investigate the effect of mTBI alone on the development of anxiety disorders. To reduce animal stress and increase the validity of our model, we employed strict stress-mitigating guidelines. These included allowing animals to acclimate to their new environment a minimum of 3 days prior to any experimental procedures, cleaning cages only once per week, minimizing handling pre- and post- surgery [28], and providing medication to alleviate pain. Similar to other experimental procedures [11], [51], animals were administered a general anesthetic prior to surgery. Our results clearly demonstrated that the increase in anxiety-like behaviors 7- and 30-days after mTBI was due to the CCI and not due to uncontrolled stress [51] or the surgery [54]. Thus, while stress may be a risk factor for the development of anxiety disorders, mTBI alone clearly caused a delayed increase in anxiety. Stress-mTBI interactions are therefore likely to mutually exacerbate the development of long-term symptoms of anxiety.

Anxiety disorders in humans have often been attributed to deficits to the GABAergic system [55]-[58] but also neuronal hyperexcitability in the amygdala [59], [60]. In agreement, we demonstrate that after mTBI, there is a significant decrease in inhibitory synaptic transmission in the BLA, including reductions in both the frequency and amplitude of mIPSCs. This reduction is consistent with the loss of GABAergic interneurons and decreased surface expression of GABA_A_ receptors, both of which are associated with increased anxiety-like behaviors [22], [61]. Our evidence that increases in anxiety-like behavior are associated with deficits to the GABAergic system in the BLA is corroborated by previous studies demonstrating that ablation of a portion of GABAergic interneurons in the BLA [22] and reduced expression of α1 and α2 GABA_A_ receptor subunits within the amygdala [62], [63] contributes to anxiogenic behaviors in animals. Moreover, the evidence that mTBI causes reductions in the surface expression of α1, β2, and γ2 subunits of the GABA_A_ receptor suggests that there might be decreases in the benzodiazepine binding site [24]. Indeed, our evidence finding reductions in GABAergic synaptic transmission and reduced surface expression of specific subunits of the GABA_A_ receptor, provide the first functional evidence that a possible reason why many patients suffering from anxiety disorders do not improve when treated with benzodiazepines [64]–[66] is because the GABAergic system is damaged, rendering these compounds less effective in mitigating anxiety-like behaviors.

Excitability within the BLA is also regulated, in part, by activation of α_7_-nAChRs [25], [67]. α_7_-nAChRs are highly expressed in the BLA [25], [44], play an important role in modulating both GABAergic [68] and glutamatergic synaptic transmission, [26], and induce both anxiolytic [69] and anxiogenic effects when activated by specific agonists [27]. While the primary role of α_7_-nAChR activation appears to be an enhancement of inhibitory synaptic transmission [68], α_7_-nAChR activation also enhances excitatory synaptic transmission [25], [26], [44], [68]. Here, we examined, for the first time, how mTBI alters α_7_-nAChR mediated enhancement of principal cell excitability within the BLA. Within 7 days of TBI, we found a significant increase in the surface expression of α_7_-nAChRs and associated increases in the current mediated by α_7_-nAChRs on principal neurons. Thus, we show that in addition to a significant loss of interneurons and reduction in the inhibitory synaptic transmission, an enhancement in the α_7_-nAChRs-mediated transmission contributed, in part, to neuronal hyperexcitability and long-lasting increases in anxiety-like behavior.

Is mTBI more than just a focal injury? We induced injury by placing a single mild focal impact to the left parietal cortex, yet pathological and pathophysiological alterations in the BLA, a brain region far from the impact site, was observed on each hemisphere. Thus, our study corroborates with the notion that controlled cortical impact is actually more than a focal brain injury [70], [71]. Following the impact, intracranial pressure changes on each side of the brain, though the contralateral side is less affected than the ipsilateral side [72]. We speculate that such mechanical alterations trigger a neuropathological cascade throughout the brain that ultimately leads to the pathological and pathophysiological alteration observed. We recently reported that within 24 hours of CCI, chemokines transcripts are upregulated and remains elevated for at least one week [30]. Subsequent inflammation takes place and may be the underlying cause for functional and morphological alterations in the BLA on both hemispheres here reported.

In conclusion, the results from this study demonstrated that a mild TBI leads to long lasting increases in anxiety-like behavior. This increase is a result of a significant loss of GABAergic interneurons, reduced surface expression of GABA_A_ receptors and inhibitory synaptic transmission, and, increases in the surface expression and function of α_7_-nAChRs, which subsequently increase excitability within principal neurons in the BLA. With the high incidence of anxiety disorders being reported after TBI [47], it is essential to understand the pathophysiological mechanisms underlying their development. Although the results described above suggests that alterations in inhibitory synaptic transmission and cholinergic transmission contribute to increased anxiety, further studies are needed to elucidate the mechanisms leading to pathological and pathophysiological alterations in the BLA after mTBI so that pharmacological therapies may be developed to ameliorate psychiatric conditions following injury.
